# Clay nanolayer encapsulation, evolving from origins of life to future technologies

**DOI:** 10.1140/epjst/e2020-000131-1

**Published:** 2020-11-16

**Authors:** Jon Otto Fossum

**Affiliations:** Laboratory for Soft and Complex Matter Studies, Department of Physics, Norwegian University of Science and Technology – NTNU, Trondheim, Norway

## Abstract

Clays are the siblings of graphite and graphene/graphene-oxide. There are two basic ways of using clays for encapsulation of sub-micron entities such as molecules, droplets, or nanoparticles, which is either by encapsulation in the interlayer space of clay nanolayered stacked particles (“the graphite way”), or by using exfoliated clay nanolayers to wrap entities in packages (“the graphene way”). Clays maybe the prerequisites for life on earth and can also be linked to the natural formation of other two-dimensional materials such as naturally occurring graphite and its allotropes. Here we discuss state-of-the-art in the area of clay-based encapsulation and point to some future scientific directions and technological possibilities that could emerge from research in this area.

The seminal research on graphene by Geim and Novoselov[1] [1] established great hopes in two-dimensional materials as for instance reflected in the Graphene Flagship project [2] – a €1 billion initiative of the European Commission – funding 150 or so, organizations. This flagship has brought graphene production up to commercial levels, with applications in electronics, photonics, sensors, composite and coating materials, energy, flexibles and wearables, aeronautics and space, all the way to biotechnological use (the latter however, with notable safety concerns [3]).

The history of graphite science prior to 2004 was reviewed by Geim [4]. Single-atom-thick crystallites of graphite, i.e. graphene, are usually exfoliated [1,2,4] from bulk graphite. Allotropes of graphite [5] include graphene, carbon nanotubes, fullerenes, diamond, and carbon cones [6] (Fig. 1). Naturally occurring bulk graphite on earth originates from carbon rich deposits that may once have been coal, peat or oil. The natural formation of graphite is due to heat and pressure alteration (metamorphisms) of these deposits occurring in rocks close to hot igneous intrusions, i.e. intrusions that form when magma cools and solidifies before it reaches the surface [7]. There are several classes of natural graphite formed in this way, such as crystalline flake graphite, amorphous graphite or lump graphite, where natural crystalline flakes are the most mined and used for industrial purposes, in addition to synthetically

[a] e-mail: jon.fossum@ntnu.no

[1] Andre Geim and Konstantin Novoselov, both of the University of Manchester in England, shared the 2010 Nobel prize of Physics for Their Work Producing and Characterizing Graphene.

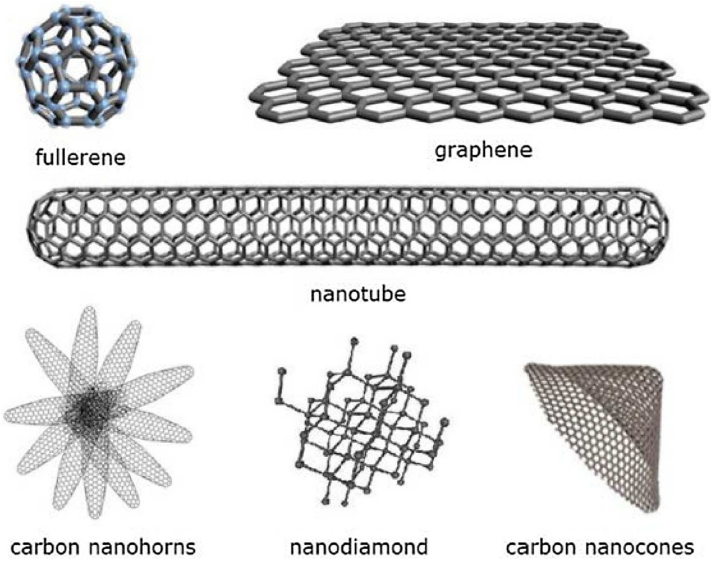

**Fig. 1.** Copied from reference [5]: Carbon allotropes.

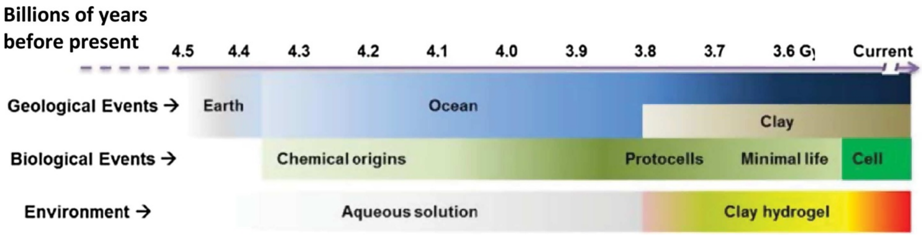

**Fig. 2.** Adopted from Supplementary Information Fig. S1 of reference [11] displaying a timeline of geological and biological events associated with early life evolution on earth approximate in billions of years before the present. The bottom row (Environment) refers to the possibility that clay hydrogels on early earth may have provided a catalytic as well as a protective confinement function for biomolecules and biochemical reactants and products.

produced graphite [8–10]. A prerequisite for the natural metamorphic formation of these nanolayered graphite structures on earth is thus presence of (i) biomass and (ii) sedimentary rocks such as clays, shales or other minerals.

Figure 2, which is adopted from reference [11] displays a timeline of geological and biological events associated with early life evolution on earth, in billions of years before the present.

Figure 3 which is copied from reference [12] displays the two-dimensional nanoscale structure of clay minerals. The generally accepted classification scheme used to describe clay nanolayered systems can be summarized as follows (for specific details see Ref. [13]) from bottom-up: Atomic sheets are forming nanolayers that in turn are forming stacked particles.

Most natural clay particles are typically of size 1 micrometer or less, composed of a stacked nanolayered type structure as drawn in Figure 3, with typically 100 layers or less in each stack [13,14]. Thus, clay two-dimensional layers come in different lateral diameters ranging from about 20 nanometers (Laponite [15] synthetic clay) via micrometers for natural montmorillonites all the way up to millimeters for natural

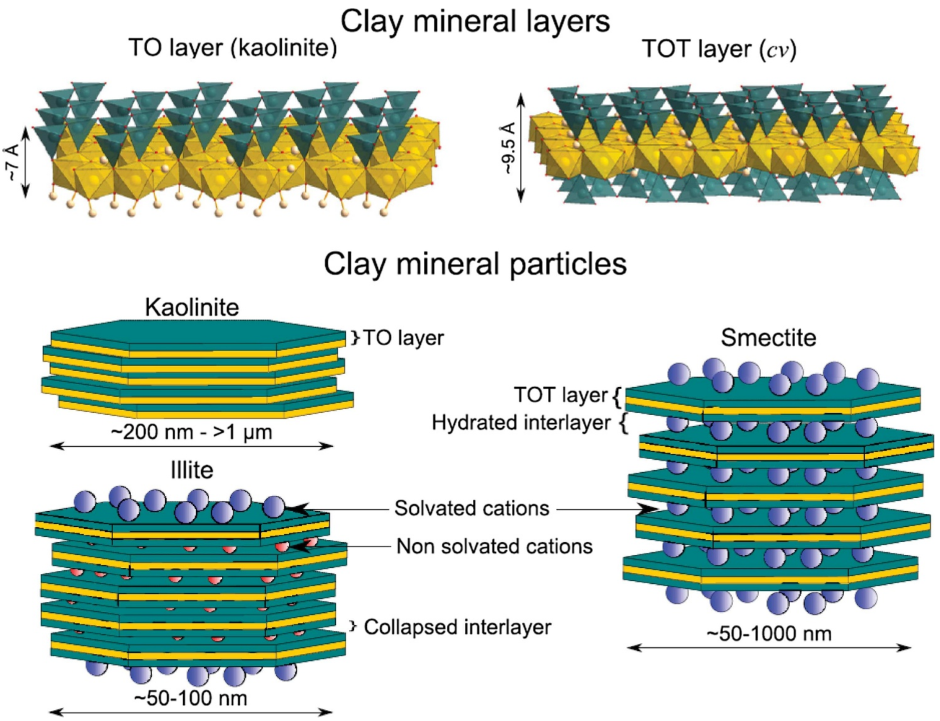

**Fig. 3.** Copied from reference [12]: Top row: Natural nanolayered clay structures. Tetrahedral-Octahedral (TO) layers (Natural kaolinite is a typical TO clay) and Tetrahedral-Octahedral-Tetrahedral (TOT) layers (Illite, and smectite clays such as natural montmorillonite, are typical TOT clays). Bottom row: Respective stacked clay mineral particles. (This figure is subject to copyright protection and is not covered by a Creative Commons license.)

vermiculites (Fig. 4b). Each of these clay nanolayers comes as of one of two types that are composed of either two or three atomic sheets:

1. one Tetrahedral sheet + one Octahedral sheet forming so-called TO-layers, or
2. one Tetrahedral sheet + one Octahedral sheet + one Tetrahedral sheet forming so-called TOT-layers.

TO-layers form strong hydrogen bonds between each other in the particle stacks and will not easily delaminate from one another [16]. Kaolinite (commonly used e.g. in porcelain, clay bricks or as paper-filler) is the prototypical example of a TO-layered clay.

Illite, which is the predominant ingredient in so-called quick clays [17], and smectite clays, e.g. natural montmorillonite (Bentonite is a commercial name for mixture of clays that predominantly consists of montmorillonite) commonly used as rheology modifiers, are both TOT clays. Very much like graphene in graphite, smectite TOT clay layers are weakly bond to each other, they can be delaminated from their parent smectite particle stacks, and they can be isolated as exfoliated single clay nanolayers. In the remainder of this text, we will focus on the TOT layers, and hereafter take the word clay nanolayer to refer to the smectite case.

Due to metallic substitutions in the Octahedral sheet, each TOT nanolayer carries a permanent negative basal surface charge of typically 1–3 electron charges per $(\mathrm{nm})^2$-area and in addition a much smaller pH-dependent charge at the edges due to hydrolysis of OH-groups thus giving a patchy nature to the clay nanolayer.

As described above, individual atomically flat polyanionic clay nanolayers may stack into plate-like macroscopic crystallites (Figs. 3 and 4), comprising macroscopic clays. The negative surface charges are compensated for by cations located in the interlayers, and they play a crucial role for the layer-layer TOT-TOT interactions, as well as for interactions with and affinity for foreign molecular species or foreign nanoparticles [12,13].

Clay properties and applications are intimately linked to their outer and interlayer surface functionalities, which can serve as catalysts for chemical reactions, as well as to their two-dimensional nanoscale character, which enables them to confine foreign species such as molecules or nanoparticles in their interlayer nano-space, thus promoting concentration of and local protection of chemical reactants and reaction products, which may result in increased efficiency of chemical reactions.

Of particular importance in this context are biomolecules and biochemical reactions. In this line, clay minerals have been proposed to play crucial roles in life's origin and evolution. This is driven by the search for experimental laboratory scale proven efficient catalytic and confinement environments for biomolecules that also would have been present at the pre-life early earth. For tutorial texts and reviews on the topic of clay minerals and the origin of life, see e.g. references [18–22].

Clay nanoscale properties as described above, combined with their wide distribution and abundance throughout earth's geological and biological timeline, including established presence on earth prior to the occurrence of the first simplest life forms (Fig. 2), have led to several works and publications suggesting roles of clays for the natural emergence of the first primitive life forms. For instance, laboratory experiments have demonstrated that it is possible to achieve synthesis of RNA from its monomers requiring only TOT clay minerals and a small amount of salt. This has consequently been suggested as a simple process for prebiotic synthesis of RNA [23]. Other laboratory experiments [24,25] have provided evidence for processes that could have formed the basis for a coevolution of clay minerals and early metabolites facilitated by sunlight photochemistry on early earth. In another type of experiments, it was demonstrated that TOT clays accelerate spontaneous conversion of fatty acid micelles into vesicles, and that TOT clay particles often become encapsulated in these vesicles. This provides a pathway for encapsulation of catalytically active surfaces within vesicles, resulting in processes that mediate vesicle replication through cycles of growth and division. This led to suggest that the formation, growth, and division of the earliest cells may have occurred in response to such interactions with clay particles and inputs of material and energy [26]. As a final example of a potential early earth clay rich environment for promoting primitive life evolution, we mention experimental laboratory studies of clay hydrogel capsules demonstrating that they provide localized concentration and protection of biomolecules and biochemical reactions [11].

As illustrated in Figures 3–5 smectite clays can occur or be prepared as two different types of basic particle entities: (i) either as particles that in effect are stacks of clay nanolayers, like graphite, or (ii) as exfoliated clay single exfoliated nanolayers, like graphene. The delamination and subsequent exfoliation of clay nanolayers, i.e. the two-step process of physically obtaining the situation depicted in Figure 4a starting from the situation depicted in Figures 4b and 4c is most often performed in aqueous suspension, and in this case, the two-step process is referred to as attractive crystalline and osmotic swelling respectively [32–34]. This delamination process can be promoted by sonification or by temperature (Fig. 5) [32]. Clays are unique in combining pronounced two-dimensional shape anisotropy, high ionic exchange, high molecular sorption capacity, and huge effective surface areas with a "patchy" functionality at the nanoscale. In terms of morphology they are the natural sibling of graphene oxide (GO). Clays are in general electrical insulators, unlike GO and

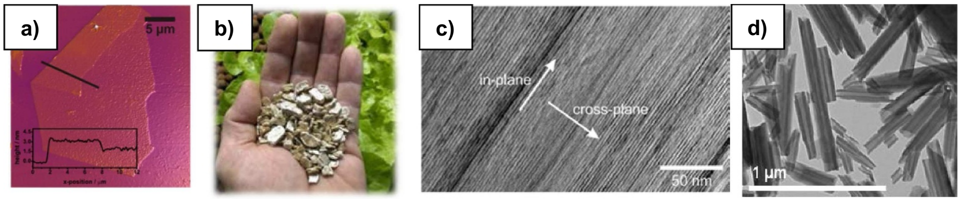

**Fig. 4.** Real examples of clay two-dimensional nanolayered-structure and lamellar morphology. (a), (b), (c) copied from reference [28] (fluorohectorite), [27] (vermiculite), [29] (fluorohectorite) respectively. (d) SEM picture taken from reference [30] of halloysite clay nanotubes, which is the clay sibling of carbon multiwalled nanotubes. Halloysite is a TO type clay that differs from kaolinite and readily accepts water in its tubular interlayer space [13,31]. (Figs. 4a–4c are subject to copyright protection and are not covered by a Creative Commons license.)

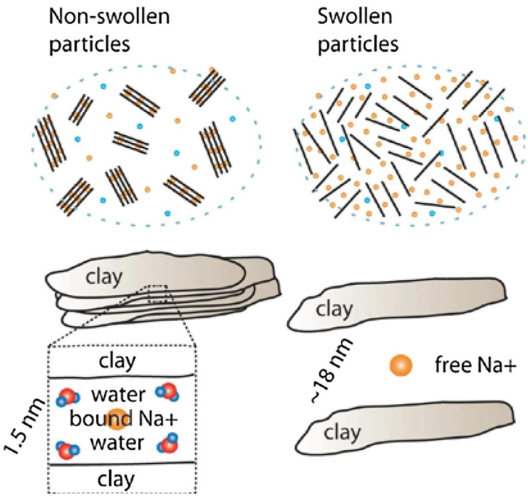

**Fig. 5.** Copied from reference [32]. Left: Non-exfoliated clay stacked particles in aqueous suspension. Right: Exfoliated clay nanolayers.

graphene. Clay nanolayers are hydrophilic like GO and unlike graphene that is considered to be hydrophobic, although this is still a debated topic [35].

In the attractive crystalline swelling situation these clays can capture foreign molecular species in the interlayer space, similar to what occurs in graphite intercalation compounds [37,38]. In the present context, we refer to this as encapsulation by intercalation.

The affinity for foreign molecules and their intercalation in the interalayer space of clays is foremost coupled to the type of charge compensating interlayer cation that is present, and secondly to the molecular interactions with the clay TOT layers themselves [13,39]. There are several cases investigated and reported in the literature with respect to capturing and retention mechanisms for encapsulation of foreign molecules inside such clay interlayers (Fig. 6), including $H_2O$ intercalation [12,13,39–46], $CO_2$ capture and retention [36,47–51], cation exchange [12,13,52], medical drug capture and release [12,13,39,53–59], capture of surfactants [12,13,39,60,61], polymers [12,13,39,62–64], or nanoparticles [65]. These encapsulation properties of clay nanolayered stacks are crucial for several engineering applications, e.g. for environmental remediation purposes [12,13,39] or for repository design for nuclear waste disposal [39,66].

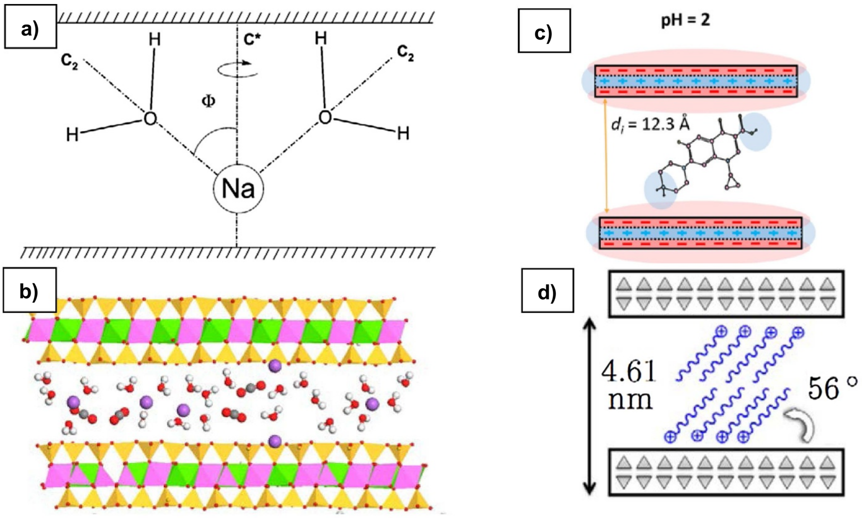

**Fig. 6.** Some examples of structural arrangements for various types of intercalated molecular species in the TOT clay interlayer space: (a) Copied from reference [43]: Schematic representation of a $Na^+$ charge compensating cation and its associated $H_2O$ molecules in the interlayer space of fluorohectorite clay. The hydration sphere, containing two water molecules, is reorienting about the C* axis, perpendicular to the clay layers. NMR spectroscopy measurements. (b) Copied from reference [36]: Schematic representation of the clay/$H_2O$/$CO_2$ system. $H_2O$ are the v-shaped molecules, $CO_2$ are the linear molecules, and the largest balls are sodium atoms. (c) Copied from reference [59]: Artistic view of interlayer capture of a ciprofloxacin drug molecule by fluorohectorite clay at $pH = 2$, which is the optimum pH for capture of this zwitterionic molecule via a cation exchange mechanism. (d) Copied from reference [61]: Suggested paraffin-type orientation of CTAB surfactant molecules in the interlayer space of fluorohectorite clay. (Figs. 6a–6d are subject to copyright protection and are not covered by a Creative Commons license.)

Now, having briefly reviewed the ability of TOT clay nanolayered stacks and TO multilayer tubes to capture, retain and deliver molecular species or nanoparticles in and from their interlayer space, like graphite can do, we move on to consider encapsulation by exfoliated clay nanolayers, in analogy with GO monolayers or graphene sheets.

Very much like exfoliated GO in aqueous suspension [67,68], exfoliated clay nanolayers in aqueous suspension form entropically driven nematic phases [13,33, 34,69–73] merely due to their individual platelet shape and thus loss of rotational degrees of freedom. It has been found that also clay particle stacks form nematic phases [14,74–78] independently of delamination and exfoliation, since also the full particles are platelet shaped, although much thicker than the individual nanolayers. In this case, due to the size of the clay particles, gravity enters as a sorting parameter for separation of nematic and isotropic phases. This gravitational effect can be used as presorting prior to delamination and exfoliation. Among parameters that influence the delamination/exfoliation process of clays as well as their nematic phases, is particle concentration that together with the confinement imposed by container dimensions controls platelet distances for the case of repulsive nematics [33,71,72,77]. Also, salinity is an essential parameter in these systems as it controls the electrostatic repulsion between the negatively charged colloidal clay particles, via the Debye screening length [79]. Combined with van der Waals attractive forces, salinity thus both commands the delamination/exfoliation process, and determines whether

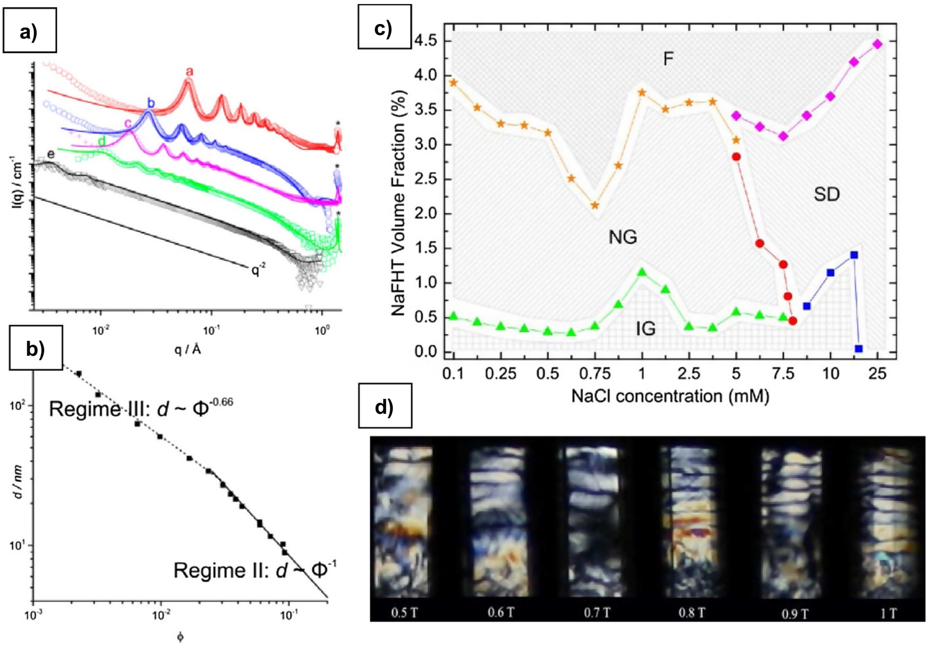

**Fig. 7.** (a–b) are copied from reference [33] where synthetic fluorohectorite clay was studied: (a) one-dimensional (1D) SAXS intensity shows a shift of the 001 reflection to higher d-spacings with decreasing concentration $\phi$. Calculated scattering curves are given by solid lines. (b) Scaling relation of the interlayer basal spacing as a function of volume fraction. The solid line corresponds to a 1D-swelling law following the equation $d = D/\phi$, the gradient in the nematic regime (dashed line) follows a distinct scaling ($d \sim \phi^{-0.66}$). (c) is copied from reference [77] and displays the salt-clay concentration phase diagram of unexfoliated clay particle nematic ordering, for a sodium-fluorohectorite system. (d) Taken from reference [81]: The so-called Fréedericksz transition with transient stripe domains observed between crossed polarizers for the same nematic system as in (c) (i.e. 3 % w/w of sodium-fluorohectorite in $10^{-3}$ M NaCl suspensions), at six different magnetic field strengths in units of Tesla (T) increasing from left to right: 0.5 T, 0.6 T, 0.7 T, 0.8 T, 0.9 T, 1.0 T. (Figs. 7a–7c are subject to copyright protection and are not covered by a Creative Commons license.)

nematically ordered clay colloids are effectively repulsive or have any attractive component [13,73,77]. Finally, entropic depletion forces [80] arising when the larger clay colloids are sterically constrained to avoid smaller particles (or molecules if present), are also important in these contexts. Container shape anchoring can control the global orientation for repulsive nematics through wall anchoring [14,76]. Clay particles respond to applied magnetic fields, and this magnetic field alignment can compete with the container induced global orientation [14,76,81,82] giving the possibility of transient stripe domain structures during the transition from wall induced alignment to magnetic field induced alignment (Fig. 7d: The so-called Fréedericksz transition).

The group of Prof. Josef Breu at the University of Bayreuth in Germany has taken clay synthesis a large step forward, by synthesizing fluorohectorite clays for which the magnitude of a well-defined and tunable sharp and homogeneous [83], layer charge allows for well controllable crystalline and osmotic swelling (Figs. 7a and 7b). The lateral dimensions of these synthetic Bayreuth clays may be hundreds of micrometers [84], which means that their aspect ratios are only surpassed by natural vermiculites [13,27]. This makes Bayreuth fluorohectorite clays particularly interesting as reliable reference systems, as well as for upscaled advanced applications.

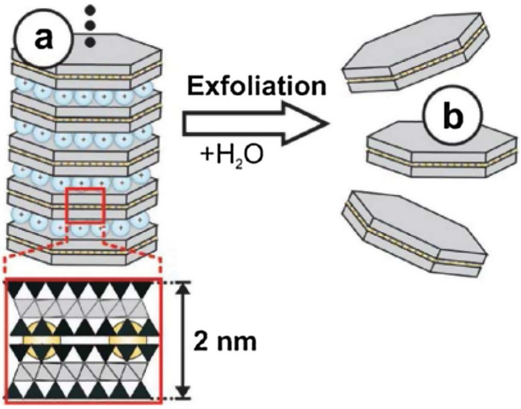

**Fig. 8.** Taken from reference [89]: Illustration of pathway developed at the University of Bayreuth for controlled ion-exchange (a) followed by exfoliation producing bi-layered TOT clay (b). (This figure is subject to copyright protection and is not covered by a Creative Commons license.)

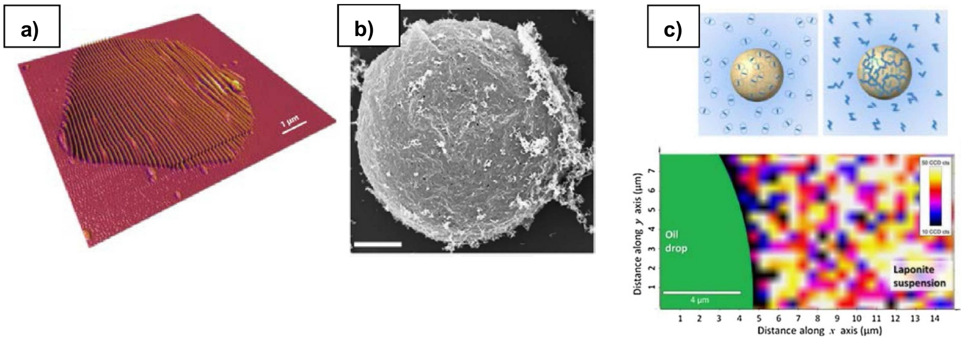

**Fig. 9.** (a) Taken from reference [85]: AFM topographical image (scale bar 2 micrometres) of a typical wrinkled delaminated clay nanoplatelet attached to a stress-released polydimethylsiloxane substrate. (b) Taken from reference [97]: SEM image of an intact clay colloidosome showing its microporous wall. Aggregated clay particles can be seen as fluffy masses adsorbed on the upper right side of the clay colloidosome. In this case colloidosomes were mechanically assembled at the interfaces of gas bubbles. Scale bar is 10 micrometres. (c) Taken from reference [95]: Salt dependent binding of clay assemblies at a liquid drop interface: The drawings are artistic views of a transition from glass- to gel-like states when Laponite clay particles are adsorbed onto a liquid drop interface at two different salt concentrations. The lower image is an experimental observation of the clay assembly at the drop interface using a confocal Raman microscope. Scale bar is 4 micrometres. (Figs. 9a–9b are subject to copyright protection and are not covered by a Creative Commons license.)

Once exfoliation has been achieved, e.g. through the suspension route outlined above, one question that arises is whether a singled-out clay mono-nanolayer can wrap objects such as droplets or nanoparticles, in the way that graphene sheets and GO nanolayers can do, see below. The first issue to address in this context is whether individual exfoliated 1 nm thin clay nanolayers are sufficiently flexible for wrapping around microscopic objects. This question is partly answered already above, simply by noting the tubular morphology of halloysite (Fig. 4d). This and other issues related to the mechanics of clay single or multi nanolayer bending is discussed theoretically in reference [86], and in reference [87] an AFM topographical image of a wrinkled delaminated clay nanoplatelet on a stress-released polydimethylsiloxane substrate was obtained, giving the in-plane elastic modulus of a singular TOT clay nanolayer (Fig. 9a), which is comparable to that of graphene or GO. Thus, clay nanolayers clearly can wrap microscopic objects. Moreover, thicker systems comprised of double-stacked or multi-stacked TOT clays, with larger bending stiffness than the single 1 nm thick nanolayers, can easily be produced following well established protocols for exfoliation routes [88,89] (Fig. 8).

Two related areas where clay wrapping has been studied and developed are for Pickering emulsion [90] stabilization [91–95] by assembling clay colloidal layers bonded to emulsion drop interfaces by capillary forces (Fig. 9c), and for fabrication of colloidosomes [96] from assemblies of clay layers [97,98] (Fig. 9b).

One particularly interesting way of assembling colloidal particles, e.g. clay colloids, on drop interfaces for clay capsule fabrication, uses electrohydrodynamic [99] driven assembly [100–102]. In this case, colloidal particles are driven by a hydrodynamic flow field that is induced by an applied DC electric field. This assembly method depends on the electrical properties of the suspending fluids (carrier fluid and drop fluid). Some examples of this are shown in Figure 10.

All experiments reported in the literature until present involve wrapping using assemblies of layered clay particles, and there is no reported case yet of wrapping using a single clay nanolayer, which due to the required lateral size would need Bayreuth type clays described above, or exfoliated vermiculite.

This is how far clay nanolayers have been taken with respect to thin single layer wrapping, and now we will turn to graphene and GO for inspiration for future work in this area:

A liquid droplet adhering to a flat solid surface deforms as it wets the surface. A droplet adhering to a thin flexible solid sheet may instead deform the sheet so that it wraps around it. The term *capillary origami* was first introduced by in 2007, see Figure 11. Macroscopic sheets can be cut into different shapes and spontaneously form a wide range of 3D structures in this way. The phenomenon persists down to the micro- and nanoscale, and molecular thin sheets such as graphene may spontaneously wrap around droplets due to capillary interactions. There is considerable activity experimentally and theoretically in this field. Capillary origami is a simple and robust means of creating 3D nanostructures from 2D sheets.

Wrinkles or crumpling occur naturally in connection with wrapping of thin sheets. This also persists at nano-scales: Molecularly thin sheets such as graphene or clay-nanolayers have typical wrinkling length of the order 100 nm and upwards (Figs. 9, 11). Observing wrinkling of sheets on solid or liquid substrates, can be a simple and efficient way to study mechanical properties of these sheets. Small wrinkles (folds) are almost always present in thin sheets subject to stress or thermal fluctuations, and this can have a profound effect on their elastic properties, as it may renormalize the effective bending rigidity by up to four orders of magnitude in graphene [109]. There is an ongoing effort to control or remove wrinkles, and it has been shown that it is possible to "iron out" wrinkles in graphene adhering to expanding surfaces [110]. Wrapping of macroscopic films can also be controlled by electric fields due to electro-wetting and direct electric forces on the film [111], which possibly can be applied to clay layers.

There is a need to develop experimental systems for controlled nano-wrapping of droplets and particles by nanolayers based on naturally occurring non-toxic materials such as clays, including electric and magnetic field-controlled wrapping, i.e. combining elasto-capillary assembly with traditional methods of manipulation of nanoscale objects. Although such combination methods have been applied to macroscopic sheets, the use of such methods to control nanolayers is far less explored. The role of degree of wrinkling/crumpling of nanolayers needs to be investigated,

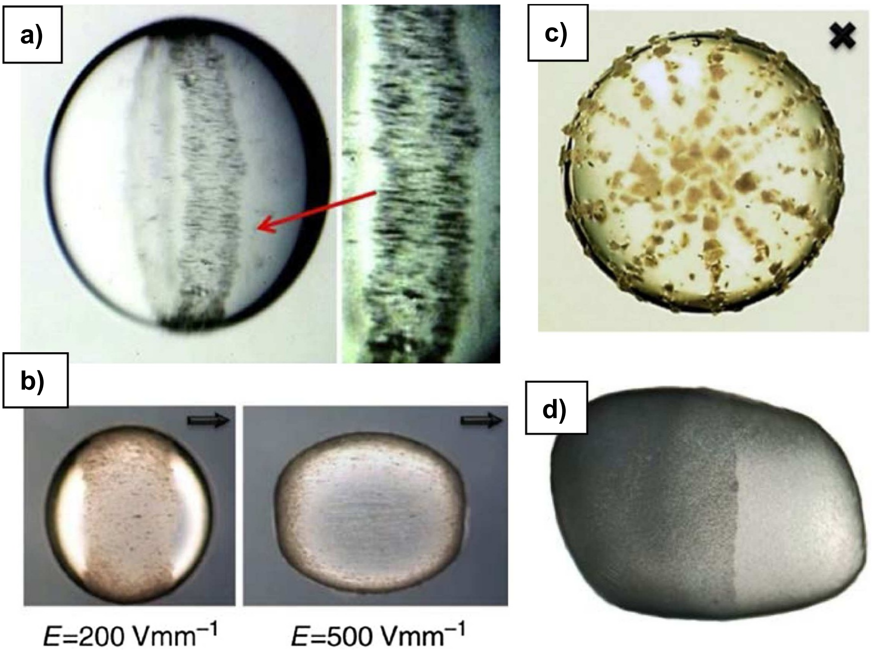

**Fig. 10.** (a)–(c) are taken from reference [101] and display clay colloidal ribbons (a,b), a clay cage (c) and a clay-based Janus capsule (d), all obtained from electrohydrodynamic driven assembly onto oildrop interfaces. The drops depicted all have diameters slightly above 1 millimeter. The direction of the applied electric DC field is horsiontal in all images, indicated by arrows in (b) and (c): (a) Perspective view of a clay ribbon film on a drop. (b) Ribbons such as in (a) made from clay colloids can be stretched to span the entire drop surface, thus closing the capsule, by utilizing the electric polarizibility of the clay particles [103] that stay confined to the drop interface by capillary forces after they are assembled there. (c) Colloidal cage formed by large fluorohectorite clay plate shaped particles. Clay chain structures are seen in the direction of the applied electric field. (d) Taken from reference [102]: Electrohydrodynamic produced Janus colloidal shell with asymmetric permeability and conductivity, where the left part of the shell is composed of polystyrene particles, whereas the right part is composed of clay particles.

understood and controlled. Although clay nanolayers wrinkle on stretched surfaces (Figs. 9, 11), there are no studies of the micro-elastic properties of freely suspended clay nanolayers, and it is not known if they crumple as much as graphene. This is essential for understanding and controlling adhesion of freely-suspended sheets to droplets e.g. for emulsion applications.

The end-purpose of encapsulation such as discussed here is targeted and controlled release of wrapped contents. Parameters that may be important for release control, are salinity or pH of surrounding suspensions, temperature, electric- magnetic- or fluid flow-fields. This is coupled to mechanical and chemical stability of capsules. For instance, the development of new bioactive agents (medical drugs) is an expensive and time-consuming process. Improving the delivery of drugs already in the market is a cheaper and faster process, which needs development of new carriers. Conventional oral drug formulations, such as tablets or capsules, usually are designed to provide immediate release of the drug after the administration with no control of release rate. This can lead to significant fluctuations in the concentration level of the drug into the organism. Control of these pharmacodynamic effects can be achieved e.g. by encapsulation, which in addition to promoting controlled release of the drug,

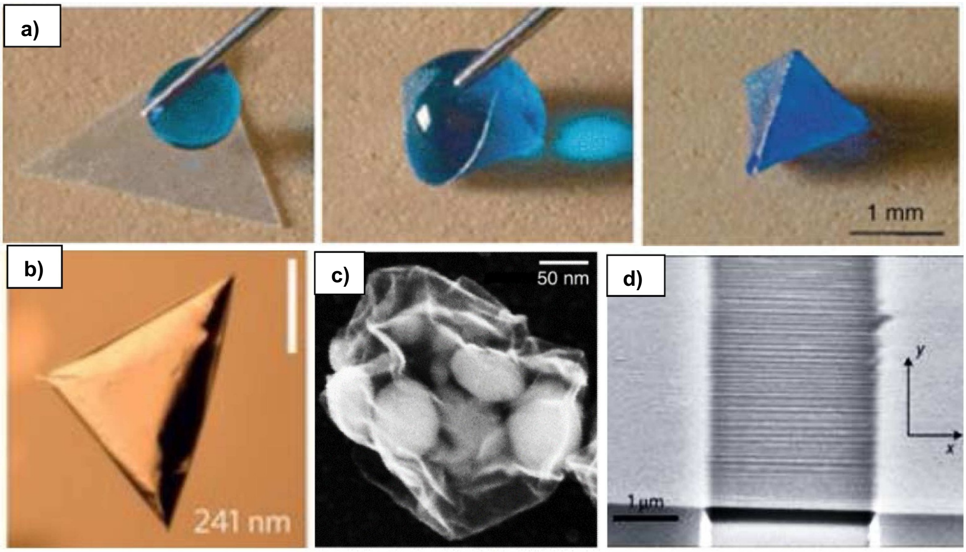

**Fig. 11.** Wrapping droplets from nm to mm length-scales: (a) (Top row) Copied from reference [104]: Spontaneous capillary wrapping of thin film on mm sized macroscopic droplet. (b) Copied from reference [105]: Wrapping macroscopic droplet (scale bar 1 mm) with a 241 nm thick film. (c) Copied from reference [106]: Graphene wrapping of nanoparticles obtained from evaporating micron-sized droplet that was spontaneously wrapped by graphene sheets. (d) Copied from reference [107]: Periodic wrinkling (ripples) of graphene seen in AFM, wavelength of around 200 nm to micrometres are typically observed, the in-plane elastic modulus of graphene is measured to approximately 1 TPa [108], compared to periodic wrinkling of the clay monolayer nanosheet in Figure 8a, with wavelength 150 nm, and in plane modulus approx. 0.2 TPa. (Figs. 11a–11d are subject to copyright protection and are not covered by a Creative Commons license.)

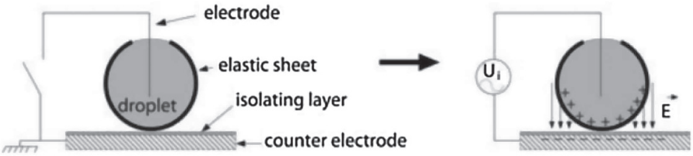

**Fig. 12.** Taken from reference [111]: Electric field-controlled wrapping and unwrapping of a thin macroscopic elastic membrane around a liquid droplet. (This figure is subject to copyright protection and is not covered by a Creative Commons license.)

can provide protection against chemical degradation (improved shelf-life), as well as increase efficiency and decrease side effects.

The bending rigidity of freestanding graphene seems to be dominated by microscopic folds in the structure, it is not entirely clear whether this is due to thermal fluctuations or “frozen” fold structures. Examples of open research questions and hypotheses related to wrapping /encapsulation by clay nanolayers may be: What is the maximum bending possible for clay? Is there a crumpled state like in graphene? A clay nanolayer (∼1 nm) is somewhat thicker than graphene (∼0.3 nm). If crumpling is weak, it opens for the interesting question if freely suspended clay nanolayers are effectively softer than graphene due to less nano-crumpling. If crumpling exist in clay nanolayers, how does it renormalize bending rigidity? This is an open question also in graphene, and it is not known which is more important; thermal fluctuation-induced, or "frozen" folds [112]. Are electric fields an efficient means to control nano-wrapping by clays? What are the possible mechanisms for this: electro-wetting, direct electric forces, electro-hydrodynamics? What are the differences between natural (e.g. vermiculite) and synthetic (e.g. Bayreuth type fluorohectorite) clay nanolayers with respect to wrapping, for instance related to the defect structure that should affect order and periodicity of the patterns? Which parameters (e.g. water salinity/pH, surfactant functionalization of colloid surfaces, temperature, applied electric-, magnetic, or hydrodynamic flow-fields) must be tuned to control efficient (with respect to time-scale and potential for up-scaling) encapsulation of drop suspensions or nano-particles in exfoliated clay nanolayers? Which parameters must be tuned in order to control efficient (with respect to time-scale and potential for up-scaling) "opening up" or degradation of capsules for release of encapsulated objects? As the clay basal surfaces are negatively charged, adhesion to nano-particles either needs an effectively positive zeta potential, or the clay surface (or the nano-particles) could be functionalized with surfactants. Currently there is obviously much interest in finding ways to capture virus nano-particles (e.g. Covid-19). Can clay layers capture virus particles? Many virus particles have a weak negative zeta potential [113], and surfactant functionalization may be needed for clay to capture such particles.

Compared to graphene, graphene-oxide and other quasi-2D materials, clay nanolayers are almost unexplored. Working on clay nanolayer bending can shed light on micro-mechanic of thin nanosheets in general. As described above, buckling experiments on monolayer clay show that they are as flexible as graphene and can easily be bent on the nanoscale. One may therefore expect clay nanolayers to have similar mechanical properties as graphene, however there might exist important differences such as types of mechanical defects and ability to crumple.

Bacteria or algae have comparable sizes to droplets or nano-particle-aggregates such as discussed here. Graphene has already been observed to wrap around bacteria. One goal for future research would be production of affordable effective materials for capture of microorganisms, or as mentioned above, maybe even virus.

Gluing soft materials has long been a technological challenge. It was recently shown that simple silica nanoparticles can glue soft gels and biological materials (liver), which offers a new direction for wound healing [114]. Using clays one can possibly aim for equivalent applications, either in using clay nanolayers in connecting large tissue wounds (micron-sized flakes) or maybe working as barriers between tissues during surgery.

Mechanical instabilities of materials are regarded as a promising route to introducing functionality in man-made structures and materials from nanometer to macroscopic scales [115]. Nature makes frequent use of mechanical instabilities to introduce functionality. Striking examples of this are the surprising connection between wrinkling and colour: The photonic structures responsible for coloration in some flowers are created by buckling of cell layers. Periodic wrinkling structures in clay nanolayers could be explored for such photonic applications. This could be coupled to works on 1D photonic crystals (Bragg stacks) based on nematic phases, so-called photonic water systems [116].

Clay minerals are natural, they are proven stable during geological timescales, they are considered safe for the environment and non-toxic [56]. This extraordinary property profile bears a large unexploited potential in materials technology. Clays are abundant materials in the environment, and will likely be easier to implement in large scale applications than for example graphene, which in addition may have toxic shortcomings [3]. Simple and robust methods for exfoliation and wrapping of single clay nanolayers are essential for large scale industrial applications.

The science described here might have a short way to real-life applications. The global micro-encapsulation market size was valued at USD 7.88 billion in

2019 [117]. Food and beverage, cosmetic, pharmaceutical, agrochemical and coating industries are main users of microencapsulation. Emulsions stabilized by clay nanolayer wrapping would likely have superior properties compared to current technologies, in for example creating impenetrable barriers for gases such as oxygen, or for microorganisms, deteriorating the encapsulated product. Although ecological food is gaining popularity, most food production rely on pesticides and fertilizers.

There is currently much effort put into reducing the use of conventional pesticides, and also in developing biocompatible, biodegradable, intelligent, and responsive agrochemical products. Microencapsulation of pesticides with clay nanolayers could minimize the risk of too rapid dissolution or volatilization of conventional pesticides and may also protect biopesticides from chemical degradation before entering the target. It was recently shown that clays (stacks) can be used for topical RNA delivery in plants for protection against virus attacks [118]. Making pH and temperature or humidity responsive encapsulations can be used in smarter agrochemicals that release content at optimal times in the growth process.

Research projects these days are often evaluated in terms of their *relevance to the UN sustainability goals*. Clay wrapping and encapsulation such as discussed here would is clearly be relevant for *Goal 2*: *Sustainable agriculture*: Helping creating encapsulation technologies would allow for a more sustainable agriculture by simple and affordable biopesticides encapsulation. *Goal 3*: *Good health and well-being for people*: Control of bacterial contamination of food products such as meat, fruit, processed food via new types of encapsulating for controlled dosing of bioactive agents in droplets (for application on external wounds or in the intestinal system). *Goal 6: Clean water and sanitation*: Biocompatible trapping of bacteria or harmful algae in water using dissolved clay nanolayers [119] could be used in sanitation applications. Goal 12: *Responsible consumption and production*: Clay wrapping, and encapsulation might replace harmful micro-/nano-plastics. Wrapping of nanoparticles and toxic volatile molecules might mitigate or help in reducing the impact of harmful substances used in industry and agriculture.

In conclusion, clays are the siblings of graphite and graphene/graphene-oxide. Clays maybe the prerequisites for life on earth. There are two basic ways of using clays for encapsulation sub-micron entities such as molecules, drops, nanoparticles, which is either by encapsulation in the interlayer space of clay nanolayered stack particles ("the graphite way"), or by using exfoliated of clay nanolayers to wrap packages ("the graphene way"). There is still a lot to do in the area of clay research, in particular regarding clay-based encapsulation as discussed here, so let's continue moving.

Open access funding provided by NTNU Norwegian University of Science and Technology (incl St. Olavs Hospital - Trondheim University Hospital). The author acknowledges all students, collaborators, and friends that have made significant contributions to the research described in this text. Special acknowledgements to Paul Dommersnes and Josef Breu for very fruitful discussions regarding the current and other topics. The following recent grants from the Research Council of Norway have supported the activities described here: 280643 (Petromaks2 Program: Nanofluids for IOR and Tracer Technology), 272919 (M-Era.Net/Nano2021 Program: Fabricating cellulose nanocomposites for structural coloration), 250619 (Nano2021 Program: Graphene-NanoClay Systems), 250728 (ClayCap – $CO_2$ Capture and Retention by Smectite Clays).

**Open Access** This is an open access article distributed under the terms of the Creative Commons Attribution License (https://creativecommons.org/licenses/by/4.0/), which permits unrestricted use, distribution, and reproduction in any medium, provided the original work is properly cited.

**Publisher's Note** The EPJ Publishers remain neutral with regard to jurisdictional claims in published maps and institutional affiliations.
